# HDAC3 as a Molecular Chaperone for Shuttling Phosphorylated TR2 to PML: A Novel Deacetylase Activity-Independent Function of HDAC3

**DOI:** 10.1371/journal.pone.0004363

**Published:** 2009-02-10

**Authors:** Pawan Gupta, Ping-Chih Ho, Sung Gil Ha, Yi-Wei Lin, Li-Na Wei

**Affiliations:** 1 Department of Pharmacology, University of Minnesota Medical School, Minneapolis, Minnesota, United States of America; 2 Institute of Microbial Technology, Chandigarh, India; UT MD Anderson Cancer Center, United States of America

## Abstract

TR2 is an orphan nuclear receptor specifically expressed in early embryos (Wei and Hsu, 1994), and a transcription factor for transcriptional regulation of important genes in stem cells including the gate keeper Oct4 (Park et al. 2007). TR2 is known to function as an activator (Wei et al. 2000), or a repressor (Chinpaisal et al., 1998, Gupta et al. 2007). Due to the lack of specific ligands, mechanisms triggering its activator or repressor function have remained puzzling for decades. Recently, we found that all-*trans* retinoic acid (atRA) triggers the activation of extracellular-signal-regulated kinase 2 (ERK2), which phosphorylates TR2 and stimulates its partitioning to promyelocytic leukemia (PML) nuclear bodies, thereby converting the activator function of TR2 into repression (Gupta *et al.* 2008; Park *et al.* 2007). Recruitment of TR2 to PML is a crucial step in the conversion of TR2 from an activator to a repressor. However, it is unclear how phosphorylated TR2 is recruited to PML, an essential step in converting TR2 from an activator to a repressor. In the present study, we use both in vitro and in vivo systems to address the problem of recruiting TR2 to PML nuclear bodies. First, we identify histone deacetylase 3 (HDAC3) as an effector molecule. HDAC3 is known to interact with TR2 (Franco et al. 2001) and this interaction is enhanced by the atRA-stimulated phosphorylation of TR2 at Thr-210 (Gupta et al. 2008). Secondly, in this study, we also find that the carrier function of HDAC3 is independent of its deacetylase activity. Thirdly, we find another novel activity of atRA that stimulates nuclear enrichment of HDAC3 to form nuclear complex with PML, which is ERK2 independent. This is the first report identifying a deacetylase-independent function for HDAC3, which serves as a specific carrier molecule that targets a specifically phosphorylated protein to PML NBs. This is also the first study delineating how protein recruitment to PML nuclear bodies occurs, which can be stimulated by atRA in an ERK2-independent manner. These findings could provide new insights into the development of potential therapeutics and in understanding how orphan nuclear receptor activities can be regulated without ligands.

## Introduction

Histone deacetylases (HDACs) are assigned to three distinct classes based on their sequence similarity to the yeast RPD3 (class I), HDA1 (class II), or Sir2 (NAD-dependent) proteins [1]–[3]. Class I includes HDACs 1, 2, 3, 8, and possibly 11 [1], [4]. Class II includes HDACs 4, 5, 6, 7, 9, and 10 [1]. HDACs generally are found in large, multiprotein corepressor complexes that are targeted to chromatin by sequence-specific DNA-binding proteins and are involved in repressing transcription [5], [6]. These DNA-binding proteins include nuclear receptors, the E-box binding proteins, and the methylcytosine-binding protein MeCP2 [6]–[8]. HDACs and histone acetyl transferases play crucial roles in regulating histone acetylation to regulate gene transcription [9]–[13].

Although the different classes of HDACs share a certain degree of sequence homology, they exhibit different specificities in various systems [14]–[17]. Class I HDACs function in developing embryos and carcinomas [18]–[22]. HDAC3, a class I HDAC [13], is usually found in corepressor complexes such as N-CoR, SMRT and RIP140 complexes [23]–[25]. A thorough characterization of the structural and functional properties of HDAC3 identified a nonconserved region in its carboxy-terminal region that is required for histone deacetylation and transcriptional repression [13], [26]. It was suggested that this carboxy-terminal domain acts in concert with the putative catalytic domain of the protein. In addition, a nuclear export signal is present in the central portion of the molecule (amino acids [aa] 180–330), and a nuclear localization signal is present in the carboxy-terminal region (aa 313–428) [26].

Although HDAC3 functions primarily in histone deacetylation [9]–[13], a growing list of its nonhistone substrates suggests a role for HDAC3 in biological processes beyond transcriptional repression [13], [27]–[29]. Studies have suggested that HDAC3 associates with complexes that are not directly involved in regulating genome activity [30], but it is not known if formation of these complexes requires its deacetylase activity. HDAC3 has been shown to function as a carrier/bridging molecule, targeting RbAp48 to the retinoblastoma protein [31]. HDAC3 also localizes to the mitotic spindle and is required for kinetochore–microtubule attachment [29]. It is presumed that most of the pathways involving HDACs require deacetylase activity [13] but this has not been demonstrated conclusively [30]. We previously demonstrated that HDACs 3 and 4 interact constitutively and directly with the orphan nuclear receptor TR2 via its DNA-binding domain [27]. We also reported that ERK2-phosphorylated TR2 is recruited to PML nuclear bodies (PML NBs) for its subsequent small ubiquitin-like modification (SUMOylation) and function as a potent transcriptional repressor [32], [33]. An important question that remains to be answered is how phosphorylated TR2 is facilitated to the PML NBs.

TR2 is specifically expressed in early embryos [34], and is a transcription factor for transcriptional regulation of important genes in stem cells including the gate keeper Oct4 [32]. It is known to function as an activator [35], or a repressor [36], [37]. Due to the lack of specific ligands, mechanisms triggering its activator or repressor function remains puzzling for decades. Recently, our finding of all-*trans* retinoic acid (atRA) triggered phosphorylation of TR2 stimulates its partitioning to PML nuclear bodies, which converts the activator function of TR2 into a repressor [32], [33], prompted us to examine how phosphorylated TR2 is recruited to PML, an essential step in converting TR2 from an activator to a repressor.

In the present study we demonstrate that the interaction of HDAC3 with TR2 can be stimulated by phosphorylation of TR2 at a specific ERK2 target. Furthermore, HDAC3 serves to target this specifically phosphorylated TR2 to PML NBs for its subsequent SUMOylation. Importantly, this novel function of HDAC3 is independent of its deacetylase activity. Finally, a novel ERK2-independent activity of atRA is identified, which stimulates translocation and nuclear enrichment of HDAC3 to form nuclear complex with PML.

## Results

### The effect of specific TR2 phosphorylation on its interaction with HDAC3 and PML

**Figure 1:**
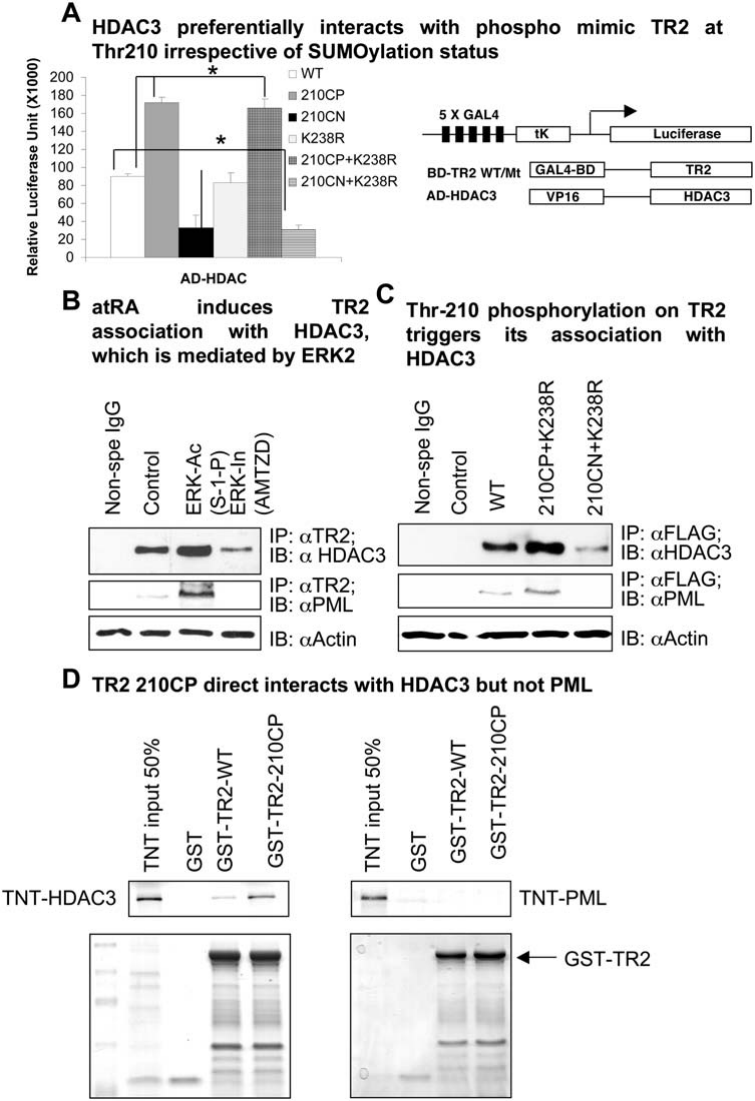
Phosphorylation of Thr-210 but not SUMOylation of Lys-238 enhances association of TR2 with HDAC3. (A) Interaction of HDAC3 with TR2 full-length (WT/Mut) constructs. A mammalian version of the two-hybrid system was used to examine *in vivo* interactions between HDAC3 and TR2 full-length (WT and phospho and/or SUMO Mut) constructs in COS-1 cells. Asterisks denote significant differences (two-tailed Student's *t* test; *, *P<*0.01). (B) The association of endogenous TR2 with HDAC3 in P19 cells was examined in the presence of reagents triggering ERK2 activation (ERK2-Ac) or inhibition (ERK2-In). (C) P19 cells were transiently transfected (0.5 μg/ml, 16 h) with FLAG-tagged WT TR2, FLAG-tagged negative SUMOylation mutation in combination with phosphomimetic (210CP+K238R) or negative phosphorylation mutations (210CN+K238R). Association with HDAC3 was examined by coimmunoprecipitation. (D) GST pull-down assay of WT/MT TR2 with effectors. Recombinant GST–TR2 WT and Thr-210 phosphomimetic mutant proteins were expressed and purified from *E*. *coli* and incubated with ^35^S-labeled *in vitro*-transcribed and translated (TNT) HDAC3 and PML (upper panels). Interacting proteins were resolved by SDS–PAGE and analyzed by autoradiography (upper panels). Half of the TNT protein sample was taken as TNT input (bottom panels). HDAC3 interaction with TR2 was increased when TR2 was phosphorylated at Thr-210 (left panel), whereas PML showed no interaction with WT or phosphomimetic TR2 (right panel).

Our recent report [33] showed that site-specific phosphorylation of TR2 at Thr-210 modulates its association with the effector molecules PCAF and RIP140, albeit indirectly by increased SUMOylation of TR2 at Lys-238. Because HDAC3 binds to TR2 at the hinge region that encompasses Thr-210 [27], we assessed its ability to mediate recruitment of Thr-210-phosphorylated TR2 to PML using a two-hybrid interaction test (Fig. 1A). The wild type (WT) TR2 interacted effectively with HDAC3 [27]. This interaction was enhanced significantly in the phosphomimetic mutant TR2 (210CP; Thr→Glu [38], [39]), but was abolished in the phosphorylation-negative mutant TR2 (210CN; Thr→Ala). Furthermore, although the SUMOylation-defective mutant TR2 (K238R; Lys→Arg) exhibited a basal level of interaction with HDAC3 similar to that of WT TR2, a double mutant containing both the phosphomimetic and the SUMOylation-negative mutations (210CP+K238R) behaved like the phosphomimetic CP210 TR2 in terms of its ability to interact with HDAC3. In contrast, a constitutive TR2 double mutant negative for both phosphorylation and SUMOylation (210CN+K238R) could not effectively interact with HDACs. These data suggest that the interaction of TR2 with HDAC3 is phosphorylation-dependent but SUMOylation-independent. This phosphorylation-dependent interaction was verified using pharmacological agents to activate or inactivate ERK (Fig. 1B). A mitogen-activated protein kinase (MAPK)/ERK activator sphingosine-1-phosphate (S-1-P) increased the association of TR2 with HDAC3, whereas addition of an ERK inhibitor 3-(2-aminoethyl)5-([4-ethoxyphenyl]methylene)-2,4-thiazolidinedione HCl (AMTZD) completely abolished the effect.

We then investigated the relationship between Thr-210 phosphorylation or Lys-238 SUMOylation and the recruitment of TR2 to endogenous HDAC3. Both WT FLAG-TR2 and the phosphomimetic and deSUMOylated FLAG-210CP+K238R TR2 double mutant associated effectively with HDAC3 (Fig. 1C). However, the ability of TR2 to associate with HDAC3 was abolished completely in the FLAG-210CN+K238R double mutant defective for both phosphorylation and SUMOylation. As predicted, the ability of TR2 to associate with PML mirrored the pattern of TR2 association with HDAC3. Thus, phosphorylation on Thr-210, but not deSUMOylation on Lys-238, triggers effective association of TR2 with HDAC3 and PML.

*In vitro* protein–protein interaction tests were undertaken to determine if the association of TR2 to the effector molecule HDAC3 was direct or indirect (Fig. 1D). TR2 was expressed and purified as a GST fusion protein. GST pull-down assays of WT and CP TR2 were carried out using *in vitro*-transcribed and translated HDAC3 or PML. WT or CP TR2 did not interact with PML (Fig. 1D, right panel), but weak interactions between HDAC3 and WT TR2 were detected; this effect was enhanced in the CP mutant (Fig. 1D, left panel). These data are consistent with the results of the cell-based assay, further supporting a phosphorylation-dependent, direct interaction between TR2 and HDAC3. This suggests that HDACs might function as a chaperone for the association of TR2 with PML, the target site for TR2 SUMOylation and its conversion into a repressor.

### A functional role for HDAC3 in targeting phosphorylated TR2 to PML

**Figure 2:**
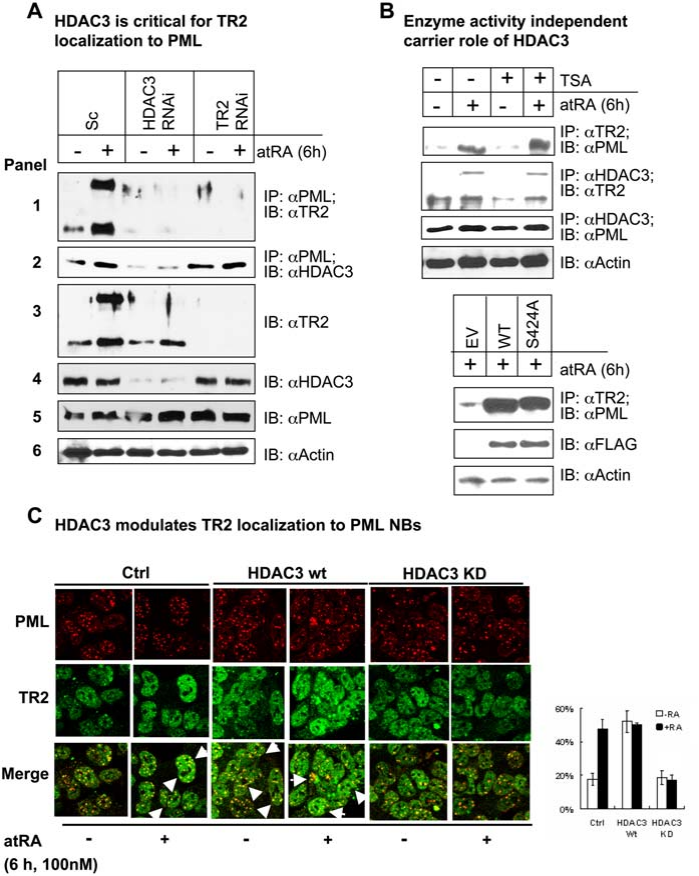
HDAC3's role as a chaperone in TR2 partitioning to PML is independent of its deacetylase activity. (A) HDAC3 modulates TR2 partitioning to PML. HDAC3 and TR2 were silenced by RNAi in embryonic stem cells and their complex formation with PML was monitored by coimmunoprecipitation. (B) HDAC3 chaperoning of TR2 to PML independent of deacetylase activity. TSA (a deacetylase inhibitor) was tested for its ability to modulate atRA-triggered TR2 and PML colocalization (upper panel). Dominant negative HDAC3 was compared to WT HDAC3 for modulation of atRA-triggered association of TR2 with PML (lower panel). (C) Immunostaining of endogenous TR2 and PML in control cells, or cells treated with atRA (0.1 μM) for 6 h in the context of gain or loss of HDAC3 expression. Nuclei were stained with DAPI. Large colocalized TR2/PML puncta in stimulated cells are marked with arrows. Right panel: Statistical analysis of the percentage of cells with colocalized TR2 and PML puncta (positive) among the total cells (positive+negative).

Because the association of TR2 with HDAC3/PML was related directly to phosphorylation on TR2 at Thr-210, and Thr-210 phosphorylation was a direct result of atRA stimulation, we monitored the role of endogenous HDAC3 in mediating atRA-triggered TR2/PML colocalization [32], [33]. SiRNA knockdown of HDAC3 (Fig. 2A, panel 4) effectively (90%) blocked the atRA-triggered association of TR2 with PML (panel 1). This was similar to the efficiency achieved by TR2 knockdown (92%; panel 3). However unlike the HDAC3 knockdown, the TR2 knockdown did not affect complex formation between HDAC3 and PML. This suggests that TR2 does not alter the direct binding of HDAC3 to PML, as reported previously [40]. However, it supports the hypothesis that HDAC3 functions as a carrier or chaperone in the mobilization of TR2 to PML.

The role of HDAC3's deacetylase activity in facilitating TR2/PML colocalization was examined using the deacetylase activity inhibitor TSA (Fig. 2B, upper panel). Interestingly, blocking the deacetylase activity of HDAC3 did not affect the atRA-triggered TR2/PML colocalization, suggesting that the carrier role of HDAC3 was independent of its deacetylase activity. However, TSA is known for many non-target effects [41], including modulation of global gene expression. We therefore sought to validate the results of these pharmacological studies by using a dominant negative mutant of HDAC3 (Ser-424→Ala) which is specifically defective in its deacetylase activity [42]. There was no apparent difference between the WT and deacetylase-negative mutant in the ability of atRA to stimulate the association of TR2 with PML (Fig. 2B, lower panel). This confirms a deacetylase-independent chaperone role for HDAC3 in stimulating TR2 localization to PML.

Immunohistochemistry was also conducted to monitor the distribution of endogenous TR2 and PML NBs in a gain- or loss-of-HDAC3-expression system (Fig. 2C). Without atRA (control cells), TR2 was only minimally (20%) colocalized with endogenous PML NBs. In the atRA-treated culture, 60% of the cells showed colocalization, as reported previously [33]. In a gain-of-function system that acquired ectopic expression of HDAC3, TR2 colocalization with PML was enhanced in both the presence and absence of atRA (60% of the cells). The enhanced, atRA-independent, increase in TR2 recruitment to PML might have been caused by saturation of the endogenous components. In contrast, in a loss-of-function system where endogenous HDAC3 was knocked down, atRA-stimulated association of TR2 with PML NBs was almost completely abolished. These results further support a functional role for HDAC3 in atRA-stimulated recruitment of TR2 to PML in this experimental system.

### Effect of ERK and atRA on complex formation of TR2/HDAC3/PML

**Figure 3:**
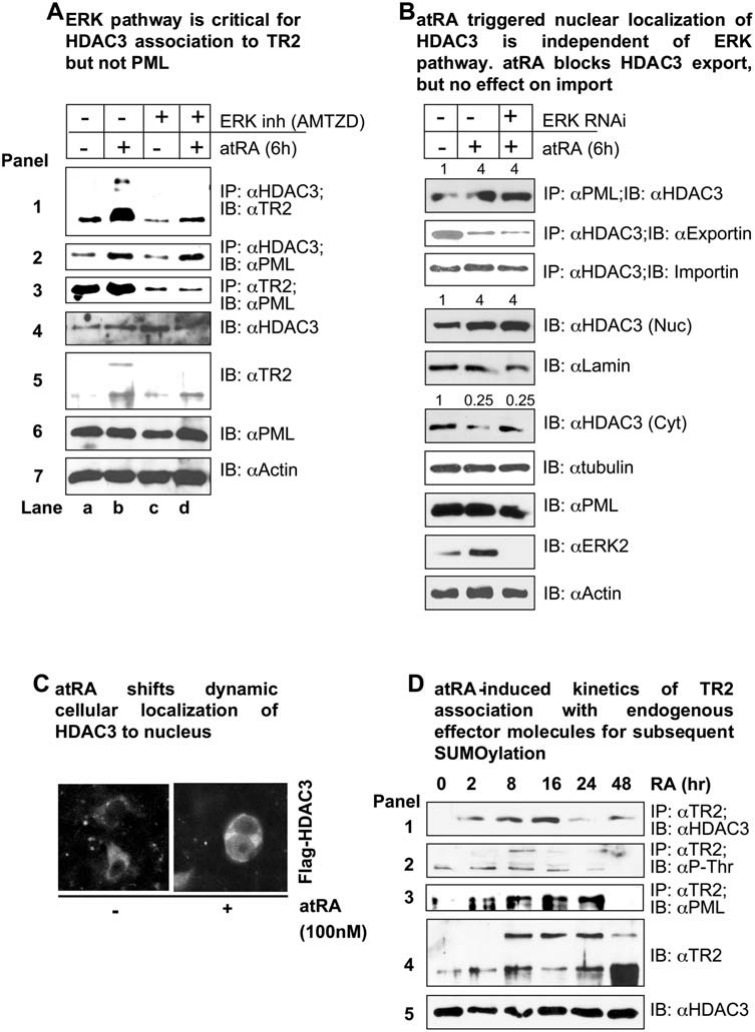
Effect of atRA and ERK on endogenous complexes with HDAC3. (A) ERK2-dependent association of HDAC3 to TR2 but not PML. ERK2 was inactivated pharmacologically and the atRA-triggered complex formation of HDAC3 with TR2 and PML was monitored. (B) Effects of atRA and ERK2 on HDAC-PML complex formation and HDAC3 subcellular distribution. Nuclear (Nuc)/cytoplasmic (Cyt) distribution of HDAC3 and its association with the import (Importin β; Karyopherin ß 1) or the export (exportin 1, CRM1) machinery were monitored in coimmunoprecipitation (IP) experiments. Immuno blot (IB) panels show all input and protein controls. Numbers above the panels indicate quantified relative values. (C) atRA-triggered nuclear enrichment of HDAC3 in P19 cells, monitored by immunocytochemistry. (D) A kinetic study of endogenous components in P19 cells after atRA treatment at 0, 2, 8, 16, 24, and 48 h (panels 1–5).

**Figure 4:**
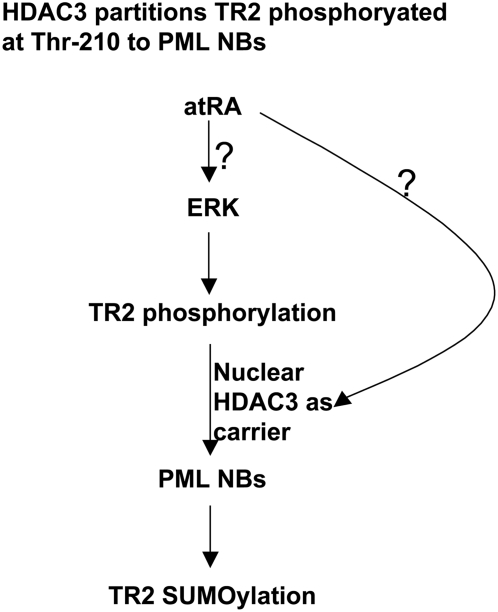
Schematic of events leading to TR2 association with PML NBs for SUMOylation. atRA activates ERK2 nongenomically, which in turns phosphorylates TR2 at Thr-210. This facilitates its recruitment to HDAC3 that shuttles TR2 to PML NBs for subsequent SUMOylation. Two major outstanding questions in this signaling pathway are pointed out with question marks.

Because HDAC3 binds both TR2 and PML, it might act as a chaperone for TR2 to PML. atRA activates ERK2, which phosphorylates TR2 at Thr-210. Phosphorylated TR2 then strongly interacts with HDAC3. To verify this model, we manipulated the experimental system with regard to two critical elements: atRA and ERK2 (Fig. 3). In the presence of an ERK2 inhibitor (AMTZD), formation of the TR2–HDAC3 complex was reduced (Fig. 3A, top panel, lane c). Furthermore, because atRA phosphorylates TR2 through the ERK pathway [33], it was unable to rescue complex formation (lane d). The direct interaction between HDAC3 and PML [40] was increased slightly by atRA treatment (panel 2, lane b), but ERK2 inhibition did not affect this interaction (lane d). In contrast, the atRA-triggered association of TR2 with PML (panel 3) was completely blocked by inhibiting ERK2 activity (lane d). Taken together, our data suggest two independent pathways. In the first, TR2 recruitment to HDAC3 is atRA-dependent and ERK2-sensitive, whereas the second pathway (in which HDAC3 recruitment to PML is slightly enhanced) is responsive to atRA but is independent of ERK2 activation (see the current working model shown in Fig. 4). It is possible that TR2–HDAC3 and HDAC3–PML could exist as two separate complexes because HDAC3–PML was formed even in the absence of TR2–HDAC3 (i.e., when the culture was stimulated with atRA but inhibited by an ERK2 inhibitor).

It is known that endogenous HDAC3 is generally distributed in equilibrium between the cytoplasm and the nucleus [26], [30]. Further, HDAC3 and PML can directly interact with each other [40] and this complex formation is not dependent upon TR2 (shown above). We therefore suspected a possibility that atRA could modulate subcellular distribution of HDAC3, thereby enhancing its nuclear distribution. To examine this possibility and to determine if this particular effect of atRA (stimulation of HDAC3-PML complex formation in the nucleus) was dependent upon the ERK2 pathway, we conducted siRNA-mediated knockdown of ERK2 and monitored subcellular distribution of HDAC3 and formation of HDAC-PML complex (Fig. 3B). It appeared that HDAC3 was indeed detected in both the cytoplasm and the nucleus in normal culture; whereas atRA treatment stimulated significant nuclear enrichment of HDAC3 (comparing fractions of Nuc and Cyt). Interestingly, this particular effect of atRA on HDAC3 nuclear enrichment was not blocked by the addition of siRNA to ERK2 (RNAi). This result suggests that the equilibrium of HDAC3 has shifted in favor of its nuclear localization, and that this phenomenon is ERK2 independent. Thus, the effect of atRA on HDAC3 nuclear enrichment is clearly different from its effect on TR2 phsophorylation that is ERK2-dependent as shown in our previous study.

To gain insights into the molecular mechanism of atRA-triggered HDAC3 nuclear enrichment, we monitored in vivo interaction of HDAC3 with exportin (the principal export machinery, CRM1) and importin-β1 (karyopherin β1) (Fig. 3B). Interestingly, in atR treated cultures, no change was detected in the interaction of HDAC3 with importin-β1 but a clear reduction was detected in its interaction with exportin 1. This suggests that atRA changes the equilibrium of HDAC3, in favor of its nuclear retention, probably due to a selective blockage of its export machinery. Other studies using immunocytochemistry has shown HDAC3 localization mainly in many fine punctate foci of the cytoplasm and in a diffused pattern in the nucleus [26], [30]. We also observed a similar nuclear and cytoplasmic pattern of HDAC3 in the control cultures, but a nucleus-enriched pattern in cultures treated with atRA (Fig. 3C). This validates the biochemical data shown in Fig. 3B. Therefore, the result showing atRA-stimulated nuclear enrichment of HDAC3 provides some explanation for the increased nuclear HDAC3-PML complex formation. However, how atRA stimulates nuclear retention, or enrichment in the nucleus, of HDAC3 remains to be investigated. This is indicated with a question mark in our current working model shown in Fig. 4.

We also examined the kinetics of endogenous complex formation at different time points following atRA treatment (Fig. 3D). The phosphorylation of TR2 at Thr-210 was rapidly stimulated, but was diminished by 16 h (panel 2). TR2 association with HDAC3 was consistently apparent at 2 h and subsided after 16 h (panel 1). The kinetics of TR2 recruitment to PML (panel 3) and its subsequent SUMOylation ([33]; panel 4) followed closely that of atRA-stimulated TR2 phosphorylation and HDAC3 association. Among the HDACs examined (HDACs 1–6), only HDAC3 showed detectable levels of expression in the P19 culture system (panel 5). The results obtained with this system that contains no foreign constructs validate our proposed model (Fig. 4): atRA stimulates TR2 localization to PML, where SUMOylation of TR2 converts it into an effective repressor of the Oct4 gene [32]. The present study has identified HDAC3 as an important carrier for the transport of TR2 phosphorylated specifically in its DNA-binding domain to PML NBs, a key step in the conversion of TR2's activity.

## Discussion

In the P19 stem cell differentiation model, atRA reduces Oct4 expression. Our previous studies reported a non-genomic mechanism by which atRA stimulates TR2 SUMOylation, a key step in the conversion of TR2 into a repressor of the Oct4 gene. In these earlier studies, we confirmed that atRA rapidly activates ERK2, leading to TR2 phosphorylation at Thr-210 and subsequent recruitment to PML NBs for SUMOylation [32], [33]. However, we failed to detect a direct interaction of TR2 with PML ([33]; Fig. 1D), leaving unanswered the means by which phosphorylated TR2 was recruited to PML. In this current study, we are able to identify this missing link: HDAC3 interacts with TR2 in an atRA-enhanced manner. More importantly, the carrier/chaperone function of HDAC3 in this pathway appears to be independent of its deacetylase activity. However, in this newly identified signaling pathway, some outstanding questions have to be addressed as pointed out in our current working model (Fig. 4). Apparently, questions remain that how atRA activates ERK2, and how atRA stimulates HDAC3 shuttling to PML at the molecular level.

HDAC3 interacts directly with the DNA-binding domain of TR2 that encompasses Thr-210 [27], and this interaction is enhanced by specific phosphorylation on Thr-210 (Fig. 1D). The exact mechanism of this enhancement remains unclear. Presumably, phosphorylation could induce conformational changes in TR2 such that it is more likely to interact with the binding domain of HDAC3. The presumed direct interaction between HDAC3 and PML [40] is increased slightly by atRA treatment, but this is ERK-independent. This remains an interesting point for further investigation. Another important finding of this study is that HDAC3 function in this pathway is independent of its deacetylase activity. Although this observation was made using the TR2 system, HDAC3 can also interact with other proteins, including many nuclear receptors that all harbor a very similar DNA-binding domain. Whether HDAC3 can have a similar role in recruiting other nuclear receptors remains to be determined.

HDAC3 associates with the nuclear dots that encompass PML NBs [40], [43], [44]. It also associates with the E3 ligase PIAS in the nuclear dots, an association that relieves the transcriptional repression exerted by HDAC3 during TFII-I mediated gene activation [44]. It is unclear whether the effect of HDAC3 in this system involves deacetylase activity [45]. Relevant to this debate, our current findings present evidence for a deacetylase-independent functional role for HDAC3 in mediating molecular interactions. The functional role of HDAC3 in leukemia has been attributed to its association with the PML–RAR fusion protein in a deacetylase-dependent fashion [13], [41]. A variety of therapeutic agents are being tested, targeting HDAC's deacetylase activity. The present study would suggest a new potential target that is independent of such activity.

## Materials and Methods

### Ethics Statement

N/A

### Plasmid Constructs and RNA interference

Mouse complementary DNAs for TR2, HDAC3, and GAL4 tk-luciferase reporter were as described previously [27], [33]. Constitutive negative/positive, point/sequential mutations involving residues Thr-210, and Lys-238 in WT TR2 (CMV/FLAG/GAL4) vector as template were made according to QuikChange XL site-directed mutagenesis kit (Stratagene) as described previously [PNAS]. HDAC3 enzymatically defective mutant Ser-424→Ala was as described previously [42]. Scrambled RNA and siRNAs for *Nr2c1*, encoding TR2 were from Dharmacon and siRNAs for *Mapk1*, encoding ERK2 were from Qiagen as described previously [33]. siRNA for *Hdac3*, encoding HDAC3 were from Qiagen 5′-AGAAGAUGAUCGUCUUCAA-3′ and 5′-GAUGAUGAUGUGUAUAAUA-3′. RNAs were introduced using DharmaFECT1 (T-2001–01, Dharmacon) or HiPerfect (no. 301704, Qiagen). Silencing was assessed by Western blots at 48–72 h. Transfection and reporter assay were as described [33], [42]. Assays were conducted at 16–36 h.

### Chemicals and Treatments

All treatments were done in Dulbecco's modified Eagle's medium containing DCC serum. atRA (0.1 μM) was added for 6 h before harvesting, unless mentioned other wise. Activators/inhibitors (Calbiochem) of ERK, sphingosine-1-phosphate (MAPK/ERK activator, 1 μM), 3-(2-aminoethyl)5-([4-ethoxyphenyl]methylene)-2,4-thiazolidinedione, HCl (ERK2 inhibitor, 25 μM), 5-(2 phenyl-py razolo[1,5-a]pyridin-3yl)-1H-pyrazolo[3,4-c]pyridazin-3-ylamine (ERK1/2 inhibitor, 1 μM) were added for 6 h. TSA (Calbiochem) [41] a deacetylase activity inhibitor, was added 1–2 h before and alongwith atRA treatment.

### TR2/PML Immunohistochemistry

Subsequent to ectopic expression/knockdown of HDAC3 (48 h), atRA treatment was done for 6 h. Cells were fixed in 4% formaldehyde and a permeation buffer, blocked for 30 mins, and incubated with primary antibodies at 4°C overnight, followed by secondary fluorescence-conjugated antibodies, at room temperature for 3 h . Images were acquired with a fluorview confocal system (Olympus). Quantification was conducted by scoring the positive cells (showing 20% colocalized PML with TR2) versus total cell numbers.

### Immunoprecipitation and Western Blot Analysis

As described previously [32], [33]. Antibodies were FLAG-M2 (F3165) from Sigma, phosphothreonine (ab-9337) from Abcam, PML (05–718) and HDAC3 from Upstate Biotechnology (05-813) and Santa Cruz Biotechnology (sc11417). TR2 (sc-9087), and PML (sc-5621) were from Santa Cruz Biotechnology. ERK2 (9108), and phosphor-p42/p44 ERK (9101) were from Cell Signaling. Exportin 1 (CRM1) and importin-β1 (karyopherin β1) were as described previously [46]

### Statistics

All statistical data were from averages of three or more independent experiments. Two-tailed Student *t* test was performed to obtain *P* values. P value<0.05 was considered to be statistically significant.
